# A Comparative Study Between Clinical Optical Coherence Tomography (OCT) Analysis and Artificial Intelligence-Based Quantitative Evaluation in the Diagnosis of Diabetic Macular Edema

**DOI:** 10.3390/vision9030075

**Published:** 2025-09-01

**Authors:** Camila Brandão Fantozzi, Letícia Margaria Peres, Jogi Suda Neto, Cinara Cássia Brandão, Rodrigo Capobianco Guido, Rubens Camargo Siqueira

**Affiliations:** 1 Escola Técnica Estadual “Philadelpho Gouvêa Netto”, São José do Rio Preto 15035-010, SP, Brazil; camila.fantozzi@gmail.com; 2 Faculdade de Medicina de São José do Rio Preto, São José do Rio Preto 15090-000, SP, Brazil; 3 Instituto de Biociências, Letras e Ciências Exatas, Universidade Estadual Paulista “Júlio de Mesquita Filho”, São José do Rio Preto 15054-000, SP, Brazil; 4 European Organization for Nuclear Research (CERN), 1211 Geneva, Switzerland; 5 Centro de Pesquisa Rubens Siqueira, São José do Rio Preto 15010-100, SP, Brazil

**Keywords:** retinal diseases, machine learning, optical coherence tomography, diabetic macular edema, artificial intelligence, support vector machine, paraconsistent feature engineering

## Abstract

Recent advances in artificial intelligence (AI) have transformed ophthalmic diagnostics, particularly for retinal diseases. In this prospective, non-randomized study, we evaluated the performance of an AI-based software system against conventional clinical assessment—both quantitative and qualitative—of optical coherence tomography (OCT) images for diagnosing diabetic macular edema (DME). A total of 700 OCT exams were analyzed across 26 features, including demographic data (age, sex), eye laterality, visual acuity, and 21 quantitative OCT parameters (Macula Map A X-Y). We tested two classification scenarios: binary (DME presence vs. absence) and multiclass (six distinct DME phenotypes). To streamline feature selection, we applied paraconsistent feature engineering (PFE), isolating the most diagnostically relevant variables. We then compared the diagnostic accuracies of logistic regression, support vector machines (SVM), K-nearest neighbors (KNN), and decision tree models. In the binary classification using all features, SVM and KNN achieved 92% accuracy, while logistic regression reached 91%. When restricted to the four PFE-selected features, accuracy modestly declined to 84% for both logistic regression and SVM. These findings underscore the potential of AI—and particularly PFE—as an efficient, accurate aid for DME screening and diagnosis.

## 1. Introduction

Artificial intelligence (AI) has emerged as a transformative technology in the field of ophthalmology, particularly in the diagnosis and management of retinal diseases. AI encompasses a variety of computational techniques that aim to mimic human cognitive processes such as learning, reasoning, and problem-solving [1,2,3]. Within AI, machine learning and its subfield, deep learning, have demonstrated significant potential in medical imaging. Deep learning, especially convolutional neural networks (CNNs), enables automated recognition of pathological features with high accuracy by extracting relevant patterns from large datasets [4,5,6,7].

In retinal imaging, AI systems have been applied to fundus photography, optical coherence tomography (OCT), and OCT angiography (OCTA) to detect and classify conditions such as diabetic retinopathy, age-related macular degeneration, and glaucoma [8,9,10,11,12,13,14,15,16,17].

Diabetic macular edema (DME), a major complication of diabetic retinopathy, is characterized by retinal thickening and intraretinal fluid accumulation due to abnormal vascular permeability. As one of the leading causes of blindness in working-age adults, DME poses a significant diagnostic and management challenge worldwide. Diagnostic tools, such as OCT, are essential, providing detailed data on macular structure, but their interpretation critically depends on the specialist’s expertise, leaving the final decision entirely in the hands of the healthcare professional. In this context, the development of AI tools to support decision-making is extremely valuable to improve patients’ quality of life and optimize clinical workflow [18].

Studies have shown that deep learning models can match or even surpass the performance of experts in identifying diabetic retinopathy from fundus images [5,18]. AI-based screening systems, such as IDx-DR, have received approval from and are being integrated into primary care settings to enable earlier diagnosis and reduce the burden on specialists [19].

Despite these advances, several limitations persist. The “black box” nature of many AI models, where the decision-making process is not transparent, raises concerns about reliability and clinical trust [20]. Furthermore, the quality and diversity of training datasets can significantly impact model performance, leading to potential biases and limited generalization across different populations and imaging devices [2,9,21]. Therefore, rigorous validation in diverse scenarios is essential.

The integration of AI into ophthalmology is particularly beneficial for regions with limited access to retina specialists. Teleophthalmology platforms incorporating AI can provide timely and cost-effective screening, aiding in the early detection of sight-threatening diseases [22,23,24,25].

Although recent literature demonstrates a growing interest in the application of AI for ophthalmological diagnoses, a systematic review revealed a specific gap: no previous study has addressed the pre-diagnosis of DME using the paraconsistent feature engineering (PFE) approach [18]. Current AI techniques focus predominantly on clinical image analysis, while PFE offers an innovative method for selecting the most informative features from raw data, boosting the accuracy of machine learning models. This work is justified by the need to explore this innovative approach, using a robust dataset to develop and compare different intelligent models that can aid in the pre-diagnosis of DME.

Thus, the main objective of this study is twofold: first, to establish which intelligent models, when combined with PFE, are most effective for DME screening; and second, to compare the performance of these models in characterizing the prediagnosis of DME accurately and reliably. By filling this gap, this research not only makes a unique contribution to the fields of ophthalmology and AI, but also aims to offer an accessible and effective tool that can improve clinical outcomes and patient quality of life.

## 2. Materials and Methods

This was a retrospective, open-label, non-randomized, comparative study conducted at the Rubens Siqueira Research Center in São José do Rio Preto, Brazil. The primary objective was to evaluate and compare the clinical analysis (quantitative and qualitative) of OCT images with a quantitative analysis performed by an AI-based software system in the diagnosis of DME. The study was approved by the Human Research Ethics Committee of the Faculdade de Medicina de São José do Rio Preto under Opinion No. 7.772.688, registered on Plataforma Brasil (CAAE: 191219925.2.0000.5415), and the Committee acknowledged and approved the request for waiver of the Free and Informed Consent Form (TCLE).

### 2.1. Study Population and Data Collection

Data from a total of 700 examinations of 387 patients with clinically suspected DME, performed between 2023 and 2024, were included in the final dataset. The study population consisted of 214 men and 173 women, with ages ranging from 23–91 years (mean age: 62.5 years). The dataset comprised 351 examinations of the right eye and 349 of the left eye. All examinations were evaluated by a specialist physician (Dr. Rubens Siqueira) and his team. The inclusion criteria were patients ≥18 years of age with suspected DME. The exclusion criteria included media opacities that significantly impair visualization, a history of allergic reactions to fluorescein dye, substance abuse.

Patients underwent a complete ophthalmologic evaluation, including best-corrected visual acuity (BCVA) using the Early Treatment Diabetic Retinopathy Study (ETDRS) protocol [26], slit-lamp biomicroscopy, applanation tonometry, indirect ophthalmoscopy, and fluorescein angiography using the Eidon FA confocal scanner (Centervue, Padua, Italy).

### 2.2. OCT Image Acquisition and Analysis

OCT imaging was performed with a Nidek RS-3000 Advance 2 optical coherence tomography scanner, which has a resolution of 7 µm and a scan speed of 40,000 A-scans per second. Acquisition protocols included macular cube scans centered on the fovea.

Structural parameters, including central subfield thickness, the presence of intraretinal fluid (IRF) and subretinal fluid (SRF), and pigment epithelial detachment (PED), were recorded. Retinal thickness was measured in micrometers and compared between manual clinical and AI-based assessments.

### 2.3. Feature Vector and Preprocessing

The AI system used a vector of 26 features for each exam. This vector was composed of:

- Demographic and clinical data: Patient ID, age, sex (male/female), and eye laterality (right/left).
- Visual acuity: Patient’s visual acuity.
- ETDRS parameters: 18 features derived from ETDRS thickness and volume maps, covering the nine macular sectors (e.g., etdrs9_1 to etdrs9_9 for thickness and etdrs9v_1 to etdrs9v_9 for volume).
- Other OCT metrics: Fovea minima (foveamin) and total area volume (whole/total).
- Diagnosis: The phenotype verified by the physician, which served as the label for supervised learning.

Table 1 details all 26 features used. Initially, the data were loaded from a structured spreadsheet. Preprocessing involved removing instances with null values to ensure data quality and integrity. To enable modeling, the categorical features (diagnosis, eye, and sex) were transformed into numerical format using the LabelEncoder encoder from the Scikit-learn Python library (version number 0.24.1).

### 2.4. Paraconsistent Feature Engineering (PFE)

A central step of the methodology was the application of PFE, an algorithm based on paraconsistent logic, to select the most relevant subset of features for diagnosis [27]. PFE evaluates the adequacy of each feature based on two independent criteria:

- α (Intraclass Similarity): Measures how similar the values of a feature are within the same class (e.g., all patients with DME).
- β (Interclass Dissimilarity): Measures how different the values of a feature are between different classes (e.g., between patients with and without DME).

**Figure 1:**
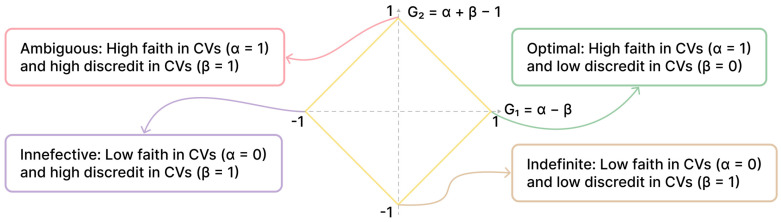
Paraconsistent plane: distribution of features by certainty and contradiction. Adapted from [27]. CV: Coefficient of Variation.

From α and β, PFE calculates two fundamental metrics: the degree of certainty (G1 = α − β) and the degree of contradiction (G2 = α + β − 1). These metrics position each feature on a “paraconsistent plane” (Figure 1). The goal is to identify features that maximize the degree of certainty (G1→1) and minimize the degree of contradiction (G2→0). This ideal point (1,0) on the plane represents a feature that is perfectly homogeneous within a class and perfectly distinct between classes, indicating high predictive power.

When applying PFE to the dataset, the algorithm identified the four most relevant features among the 24 analyzed: (ID and diagnosis were not included in the test).

1. ‘R/L eye’: The laterality of the examined eye.
2. ‘etdrs9v_7’: The volume of the external nasal ring.
3. ‘sex’: The patient’s sex.
4. ‘etdrs9_6’: The thickness of the superior external ring.

This subset of four features was then used to train and test a separate set of AI models, allowing for direct comparison with models trained with the full set of 26 features.

### 2.5. Artificial Intelligence Models

The AI system used multiple supervised learning classifiers to assess the diagnosis of DME. The models were implemented in Python (version number 3.8.8), using libraries such as Pandas for data manipulation and Scikit-learn for machine learning algorithms and metric evaluation.

The following models were evaluated:

- Logistic Regression (LR): A linear classifier commonly used in medical diagnosis due to its interpretability and effectiveness in binary classification tasks [28].
- Support Vector Machines (SVM): A robust algorithm that finds an optimal hyperplane to separate data into classes. It is particularly effective in high-dimensional spaces and for nonlinear problems when combined with kernel functions [29].
- K-Nearest Neighbors (KNN): A nonparametric method that classifies a new sample based on the majority class of its ‘k’ nearest neighbors in the feature space. It is intuitive and useful when the relationship between variables is complex and nonlinear [29].
- Decision Trees (DTREE): Highly interpretable models that use a hierarchical tree structure to make decisions, dividing the feature space into homogeneous subsets [30].

### 2.6. Experimental Scenarios and Performance Evaluation

The tests were carried out in two different scenarios to evaluate the performance of the models at different levels of diagnostic complexity:

- Scenario 1 (Binary Classification): This task classified the scans into two categories: Y (Yes), for patients with DME, and N (No), for patients without DME. This scenario included 131 positive cases (Y) and 569 negative cases (N).
- Scenario 2 (Multiclass Classification): A more complex task with six phenotypes: Y (Yes, with DME), Y-Mer (Yes, with epiretinal membrane), Y-Perifoveal (Yes, with perifoveal edema), N (No), N-Anomalies (No, but with other anomalies), and N-Mer (No, but with epiretinal membrane).

In both scenarios, the four AI models (SVM, KNN, DTREE, LR) were trained and tested with two configurations of the feature: (i) the full set of 24 features and (ii) the subset of four features selected by PFE. This resulted in a total of 16 distinct tests. Cross-validation was employed to ensure the robustness of the results and avoid overfitting.

Performance was evaluated using a comprehensive set of metrics, including confusion matrix, accuracy, sensitivity, specificity, positive predictive value (PPV), negative predictive value (NPV), F1-Score, and the area under the receiver operating characteristic curve (AUC-ROC).

### 2.7. Statistical Analysis

All statistical analyses were conducted using Python (version number 3.8.8) libraries such as Scikit-learn for metric calculations and Matplotlib/Seaborn for visualization. The analyses followed standard machine learning practices, including splitting the data into training and testing sets to assess the models’ generalization ability.

## 3. Results

Demographic Data

The study population included 700 examinations from 387 different patients. Of these, 214 (55.3%) were men and 173 (44.7%) were women. The mean age was 62.5 years (range: 23–91 years). The distribution of examinations was 351 (50.1%) for the right eye and 349 (49.9%) for the left eye.

Scenario 1: binary classification (presence vs. absence of DME)

In the binary classification scenario, the objective was to distinguish between exams with DME (representing class ‘Y’ with *n* = 131 cases), and those without DME (representing class ‘N’ with *n* = 569 cases). The results of the four models with the full set of 24 features and with the subset of four features of the PFE are presented in Table 2.

With 24 features:

The SVM and KNN models performed best, both achieving 92% accuracy. They also had AUC-ROC scores of 81.8% and 82.0%, respectively, indicating excellent discrimination ability.

The LR model also showed robust performance, with an accuracy of 91% and a good balance between sensitivity (93%) and specificity (90%).

The DTREE model had the lowest performance among the four, with an accuracy of 86%.

With four features (PFE):

With the reduced feature set, all models experienced a drop in performance.

SVM and LR were the best models in this scenario, both with an accuracy of 84%. However, sensitivity was notably lower compared to using 24 features, especially for SVM (64%).

KNN and DTREE performed even worse, with accuracies of 77% and 76%, respectively.

**Figure 2:**
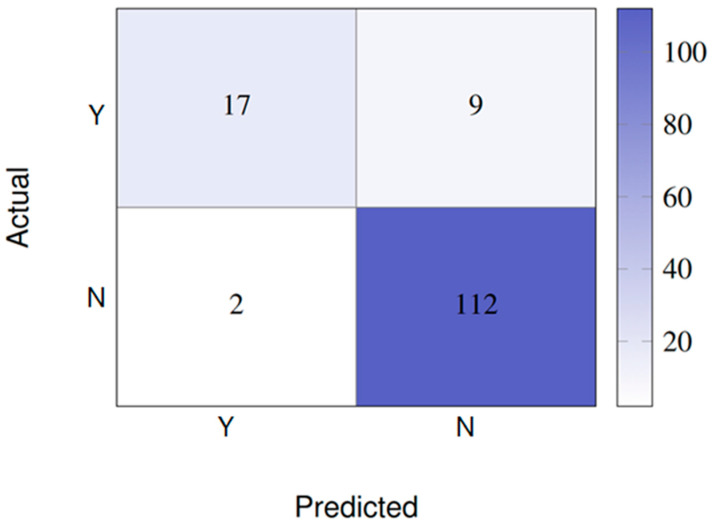
Confusion matrix for SVM/KNN with 24 features (binary scenario). This matrix shows that of the 26 actual edema cases (Y), 17 were correctly predicted, while nine were classified as negative (false negatives). Of the 114 negative cases (N), 112 were correctly predicted and only two were incorrectly classified as positive (false positives) [18].

The confusion matrix for the best model (SVM/KNN with 24 features) is shown in Figure 2, showing high accuracy in classifying negative cases (N), but with some false negatives for positive cases (Y).

Scenario 2: Multiclass classification (six phenotypes)

In Scenario 2, the models were challenged to classify the exams into six distinct phenotypes. The increased complexity of this task resulted in an overall decrease in performance compared to the binary scenario, as detailed in Table 3.

**Figure 3:**
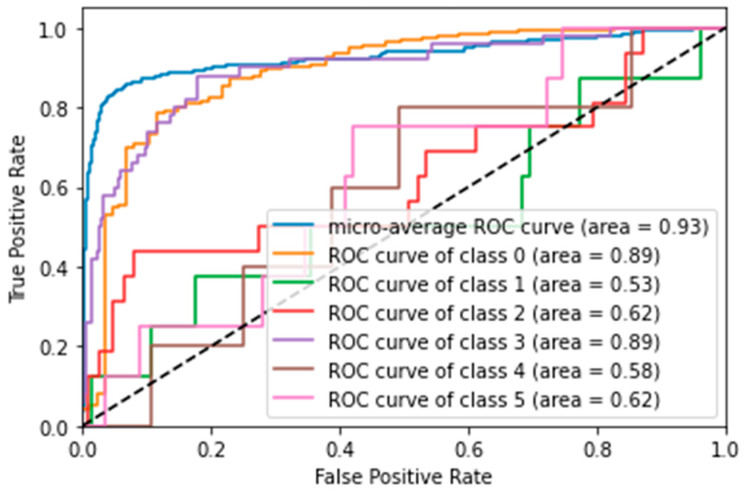
ROC curve for SVM with 24 features (multiclass scenario). Displays micro-average and per-class AUC scores, illustrating the model’s ability to differentiate multiple phenotypes.

With 24 features:

- SVM was the best-performing model, achieving an accuracy of 84.3% and an AUC score of 82.7%. ROC curve analysis (Figure 3) showed that the model was particularly good at distinguishing the ‘N’ (No: AUC = 0.89) and ‘Y’ (Yes: AUC = 0.89) classes, but struggled with less frequent classes, such as ‘N-Anomalies’ (AUC = 0.53).
- LR also performed well, with an accuracy of 81%.
- KNN and DTREE had accuracies of 80.7% and 68.6%, respectively.

**Figure 4:**
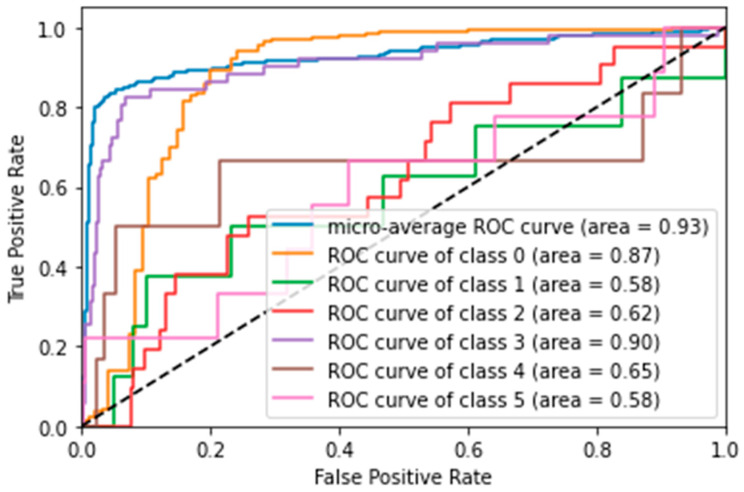
ROC curve for SVM model with 4 PFE features (multiclass scenario). Performance curve of the paraconsistent model, highlighting improvement in Y class detection and overall reduction in accuracy for rare classes.

With four features (PFE):

- Again, performance decreased with the reduced feature set. LR was the best model, with an accuracy of 78%, closely followed by SVM with 77%.
- ROC curve analysis for SVM with four features (Figure 4) showed that the discrimination ability for the ‘Y’ class improved slightly (AUC = 0.90), but overall, the performance remained inferior to the model with 24 features.

## 4. Discussion

The results of this study contribute to the growing body of evidence supporting the application of AI in the diagnosis of retinal diseases, particularly DME [7,31,32,33]. The observed diagnostic accuracy of up to 92% using the SVM and KNN models highlights the potential of AI-based algorithms to complement traditional clinical assessments [5,20]. These results are in line with previous studies, in which deep learning models demonstrated expert-level performance in identifying retinal pathologies in fundus photographs and OCT scans [4,24,34].

A notable aspect of this study is the use of paraconsistent logic to select features that increase diagnostic accuracy. This approach, combined with machine learning algorithms such as LR and SVM [35,36], allows for a robust assessment of key variables in OCT data. Previous research suggests that ETDRS-based metrics, in particular the paraconsistent algorithm, are among the most reliable predictors of visual outcomes in retinal diseases [25,37].

The difference in sensitivity and specificity between the LR and SVM models observed in this study can be attributed to inherent differences in how these algorithms handle data variance and complexity. LR models are often more interpretable and perform well on binary classification tasks when the data are linearly separable, while SVMs can be more effective in complex, nonlinear spaces but may underperform when the training dataset is limited or imbalanced [20,24].

These findings also highlight the importance of dataset quality and size in training AI models. As noted by Gulshan et al. [5] and Ting et al. [24], AI performance in ophthalmology is highly dependent on the diversity and volume of training data. The relatively small dataset of this study may limit the generalizability of the results. Therefore, larger, multiethnic datasets acquired from diverse imaging systems are essential to improve model performance and ensure clinical applicability [21,24,38]. The use of PFE not only reduces the number of features, but also improves model interpretability, which is critical for clinical adoption.

Furthermore, integrating AI into clinical workflows must address concerns related to the “black box” problem, in which clinicians are unable to identify how AI systems arrive at a diagnosis. This challenge has led to calls for explainable AI, in which model decisions are transparent and justifiable, especially in medical settings [18,20]. Furthermore, ethical considerations such as data privacy, informed consent, and bias mitigation must be addressed as AI becomes more prevalent in healthcare [1,19].

This study reinforces the usefulness of AI as a complementary tool for diagnosing retinal diseases. With further development, validation, and integration, AI systems could play a significant role in expanding access to retinal care, improving diagnostic accuracy, and supporting clinical decision-making, particularly in settings where retinal specialists are scarce.

This comparative study demonstrates that AI-based diagnostic systems using algorithms such as SVM and LF can identify DME with high accuracy, reaching up to 92% in the binary classification scenario. PFE proved to be a viable strategy for reducing the dimensionality of the problem, creating simpler and more efficient models. Although accuracy is slightly reduced, the ability to achieve reasonable performance (84% accuracy) using only four features instead of 24 offers a practical and cost-effective solution for clinical DME screening, especially in resource-limited settings.

From a clinical ophthalmological perspective, the use of AI-based systems for DME diagnosis can represent an important tool for supporting medical decision-making, especially in screening and primary care settings. The ability to achieve high accuracy with a reduced number of variables reinforces its practical applicability and cost-effectiveness. However, its safe integration into clinical routine will require multicenter validation, therapeutic impact analysis, and transparency in the interpretation of results.

## 5. Conclusions

This study demonstrates that AI-assisted models, especially when optimized via PFE, can offer accurate and cost-effective tools for DME screening. These tools are especially valuable in primary care or underserved regions. Further multicenter validation and explainable AI integration will be essential for routine clinical adoption.

## Figures and Tables

**Table 1:** Feature set used in AI-based analysis of OCT for DME diagnosis.

Order	Abbreviation	Meaning
1	ID	Patient ID
2	R/L eye	Definition of the examined eye (right or left)
3	VisualAcuity	Patient’s visual acuity level
4	etdrs9_2	Upper inner ring
5	etdrs9_4	Lower inner ring
6	etdrs9_6	Upper outer ring
7	etdrs9_8	Lower outer ring
8	foveamin	Measurement of the fovea minima
9	etdrs9v_2	Upper inner ring volume
10	etdrs9v_4	Lower inner ring volume
11	etdrs9v_6	Upper outer ring volume
12	etdrs9v_8	Lower outer ring volume
13	whole/total	Measurement of the volume of the total area
14	Diagnosis	Phenotype verified by doctor
15	Sex	Patient sex (male or female)
16	etdrs9_1	ETDRS ring center
17	etdrs9_3	Internal nasal ring
18	etdrs9_5	Internal temporal ring
19	etdrs9_7	External nasal ring
20	etdrs9_9	Outer temporal ring
21	etdrs9v_1	ETDRS ring center volume
22	etdrs9v_3	Inner nasal ring volume
23	etdrs9v_5	Internal temporal ring volume
24	etdrs9v_7	External nasal ring volume
25	etdrs9v_9	Temporal outer ring volume
26	Age	Patient’s age on the day of the examination

**Table 2:** Performance metrics for binary classification models (DME presence vs. absence) using 24 and 4 features.

Model	Features	Classification Score	Accuracy (%)	Sensitivity (%)	Specificity (%)	PPV (%)	NPV (%)	Y: F1 Score	N: F1 Score	AUC Score (%)
SVM	Normal (24)	129	92	89	93	65	98	76	95	81.8
SVM	Paraconsistent (4)	117	84	64	85	27	96	38	91	61.7
DTREE	Normal (24)	120	86	64	90	54	93	58	91	73.4
DTREE	Paraconsistent (4)	106	76	33	84	31	86	32	85	58.3
KNN	Normal (24)	129	92	89	93	65	98	76	95	82
KNN	Paraconsistent (4)	108	77	35	84	27	89	30	86	57.7
LR	Normal (24)	127	91	93	90	54	99	68	95	76
LR	Paraconsistent (4)	117	84	67	85	23	97	34	91	60

PPV: positive predictive value; NPV: negative predictive value; AUC: area under the receiver operating characteristic curve; SVM: support vector machines; DTREE: decision trees; KNN: K-nearest neighbors; LR: logistic regression.

**Table 3:** Multiclass classification performance (six DME phenotypes) using full and PFE-reduced feature sets.

Model	Features	Classification Score	Accuracy (%)	Sensitivity (%)	Specificity (%)	PPV (%)	NPV (%)	Y: F1 Score	N: F1 Score	AUC Score (%)
SVM	Normal (24)	118	84.3	68	83	81	99	74	93	82.7
SVM	Paraconsistent (4)	108	77	88	77	33	99	48	86	64.6
DTREE	Normal (24)	96	68.6	43	82	29	88	34	85	53.8
DTREE	Paraconsistent (4)	85	61	38	75	29	77	32	76	48.9
KNN	Normal (24)	113	80.7	76	84	76	95	76	89	58.5
KNN	Paraconsistent (4)	108	69	60	74	29	89	39	81	47.3
LR	Normal (24)	114	81	86	81	57	100	69	89	72.8
LR	Paraconsistent (4)	117	78	80	78	38	99	52	87	56

PPV: positive predictive value; NPV: negative predictive value; AUC: area under the receiver operating characteristic curve; SVM: support vector machines; DTREE: decision trees; KNN: K-nearest neighbors; LR: logistic regression.

## Data Availability

The original contributions presented in this study are included in the article. Further inquiries can be directed to the corresponding author(s).
